# Safety and Benefit Of Sentinel Lymph Nodes Biopsy Compared to Regional Lymph Node Dissection in Primary Vulvar Cancer Patients Without Distant Metastasis and Adjacent Organ Invasion: A Retrospective Population Study

**DOI:** 10.3389/fonc.2021.676038

**Published:** 2021-07-26

**Authors:** Weili Zhou, Yang Bai, Yangyang Yue

**Affiliations:** ^1^ Department of General Surgery, Shengjing Hospital, China Medical University, Shenyang, China; ^2^ Department of Health Management, Shengjing Hospital, China Medical University, Shenyang, China

**Keywords:** regional lymph node dissection, sentinel lymph node biopsy, surgery, tumor size, vulvar cancer, age, invasion depth

## Abstract

**Background:**

The safety and benefit of sentinel lymph node biopsy (SLNB) compared with regional lymph node dissection (RLND) and no lymph nodes removed (NA) in patients with vulvar squamous cell cancer (VSCC) was not well studied.

**Methods:**

A retrospective analysis on VSCC patients without distant metastasis and adjacent organ invasion from the Surveillance, Epidemiology, and End Results Program database between 2004 and 2016 was carried out. Within subgroups stratified by negative (LN−) or positive (LN+) regional lymph node findings, inverse probability weighting (IPW) adjusted multivariate Fine-Gray compete risk (CR) model and accelerated failure time (AFT) model was used to investigate the factors associated with and cancer-specific survival (CSS) and overall survival (OS).

**Results:**

Of the 3,161 VSCC patients treated with surgery, 287 (9.1%) underwent SLNB, 1,716 (54.3%) underwent RLND, and 1,158 (36.6%) had no regional lymph nodes removed. As illustrated by IPW adjusted multivariate regressions, SLNB was significantly associated with prolonged CSS (LN−, adjusted sub-proportional hazard ratio [sHR] = 0.42; 95% confidence interval [CI], 0.19–0.93; *P=*0.032; LN+, adjusted sHR = 0.29; 95% CI, 0.16–0.54, *P<*0.001) and OS (LN−, adjusted time ratio [TR] = 1.38; 95% CI, 0.82–2.32; *P=*0.226; LN+, adjusted TR = 2.68; 95% CI, 1.73–4.14; *P<*0.001), although the effect of SLNB on OS was not significant within the LN− cohort. Moreover, SLNB led to improved CSS (adjusted sHR = 0.40; 95% CI, 0.23–0.70; *P =* 0.001) and OS (adjusted TR=1.15, 95% CI 0.76-1.73, *P=*0.279) compared with NA. Age was a significant prognostic factor of CSS and OS, whereas tumor size, surgery type, and invasion depth were not.

**Conclusions:**

SLNB leads to significantly prolonged CSS and OS in VSCC surgery patients without distant metastasis and adjacent organ invasion than RLND, except for the similar OS in the LN− cohort. SLNB could be carried out preferentially for VSCC surgery patients without distant metastasis and adjacent organ invasion, irrespective of tumor size, surgery type, invasion depth, and regional lymph nodes metastasis. Further prospective clinical trials are warranted to confirm the findings of this study.

## Introduction

Vulvar cancer is a rare malignancy that accounts for about 5% of all gynecologic cancer cases, with more than 6,100 newly diagnosed cases yearly in the United States, leading to nearly 1,400 deaths (1). Ninety percent of vulvar cancers are squamous cell carcinomas (VSCC) (2, 3). Currently, the primary treatment for VSCC is surgical resection and radiotherapy (with or without chemotherapy) if necessary (2, 4).

Vulvar cancer usually spreads to regional lymph nodes, such as the inguinal, femoral, or pelvic lymph nodes. The more the regional lymph nodes were involved, the worse the long-term survival was (5, 6). Therefore, regional lymph nodes dissection (RLND), covering inguinal and femoral lymph nodes, was usually performed to remove lymph nodes for work-up or therapy intent. However, RLND has a high probability of short- and long-term complications that are the leading cause of death after surgical treatment, such as wound breakdown, wound infection, lymphoceles, lymphedema, cellulitis, and erysipelas (7). After implementing several new surgical techniques of lymph nodes dissection procedure, the morbidity of complications after RLND decreased in recent years but remains high and clinically meaningful (8). Thus, sentinel lymph nodes biopsy (SLNB) was preferred to replace RLND for selected VSCC patients because of its less aggressiveness and lower probability of surgery complications (9). Moreover, SLNB has been proven to have high sensitivity of more than 95% to indicate positive regional lymph nodes and a specialty of as high as 100% (9, 10). However, more than 50% of VSCC patients still received RLND alone because of the limited application of SLNB (7), which was led to by the limited evidence on the safety and effectiveness of SLNB because of the rarity of vulvar carcinoma.

Thus, it is urgent to identify the safety and efficacy of SLNB in VSCC surgery patients, especially in those with negative regional lymph node findings. Therefore, we compared the long-term survival between patients who underwent SLNB and those who underwent RLND or NA in a large real-world cohort, controlling for several factors.

## Materials And Methods

### Data Source and Study Population

The Surveillance, Epidemiology, and End Results (SEER) Program database of the National Cancer Institute was retrieved to identify patients with primary vulvar carcinoma from 2004 to 2016. Patients with the International Classification of Diseases for Oncology, 3rd Edition (ICD-O-3) primary site code of C51.0, C51.1, C51.2, C51.8 C51.9, and the ICD-O-3 histology codes of 8050-8084 (as squamous cell carcinoma) were enrolled (11).

Moreover, patients were excluded by the following criteria: 1) not squamous cell carcinoma; 2) not the first primary tumor; 3) survival months <1; 4) age at diagnosis < 18 or >80 years; 5) tumor size <1 millimeter; 6) no surgery performed; 7) debulking; 8) surgery not otherwise specified; 9) surgery performed unknown; 7) Distant metastasis; 8) Adjacent organ invasion; 9) AJCC pathologic staging criteria violation; 10) Lymph nodes removed unknown; 11) Positive lymph nodes without dissection. In addition, Debulking was excluded because it is performed for palliative rather than curative intent.

The region, insurance status, year of diagnosis, age at diagnosis, race, marital status, primary site, pathological grade, tumor size, invasion depth, surgery type, radiotherapy, lymph node size, and SLNB were derived from the corresponding fields of the SEER database. And then, they were included in regressions because they were found to be prognostic factors (12–15).

### Outcomes

Vulvar cancer-specific survival (CSS) was the primary outcome calculated according to patients whose death were attributable to vulvar cancer. In contrast, patients who died of other causes rather than vulvar cancer were considered as compete-risks. Overall survival (OS) was the secondary outcome.

### Statistical Analysis

The inverse probability weighting (IPW) was applied to adjust for the imbalance between groups. The region, insurance status, year of diagnosis, age, race, marital status, primary site, pathological grade, tumor size, invasion depth, surgery, radiotherapy, and lymph node size were all included in logit regression models to calculate the probability of the receipt of SLNB. Moreover, the IPW weights were calculated based on the pre-calculated logit models. We calculated IPW weights within each subgroup stratified by microscopically confirmed (positive histology) regional lymph nodes status—negative (LN−) or positive (LN+) regional lymph nodes findings. To assess the non-inferiority of SLNB compared with no lymph nodes removed (NA), regressions with IPW adjustment for SLNB *versus* NA were also performed. Sensitivity analysis on the missing value of lymph size was carried out to assess the consistency of the effect of SLNB *versus* RLND because clinical lymph node status was an essential confounding factor associated with the choice of SLNB and RLND.

The Kaplan-Meier survival curves were plotted and compared by the Cox test because of IPW adjustment. Multivariate accelerated failure time (AFT) regression models and Fine-Gray compete-risk (CR) models were applied to calculate the time ratio (TR) and sub-distribution hazard ratio (sHR) and their corresponding 95% confidence intervals (95% CIs). A larger TR indicates a more prolonged survival. Univariate regression models were not performed because all the abovementioned factors were included in the multivariate model without stepwise variable filtering. After all, variable filtering based on *P* value was highly controversial. Multivariate Cox regression models were also carried out to evaluate OS, but the proportional hazard hypothesis was violated for some variables. Thus, a series of AFT models using either Gamma, Lognormal, or Weibull distribution was carried out. Finally, the AFT model with lognormal distribution was chosen because it is the simplest model that best fits the sample data set.

A two-tailed *P* value of less than 0.05 was considered statistically significant. All the statistical processes were performed in STATA 16.0 software (StataCorp, College Station, Texas).

## Results

### Baseline Characteristics

**Figure 1:**
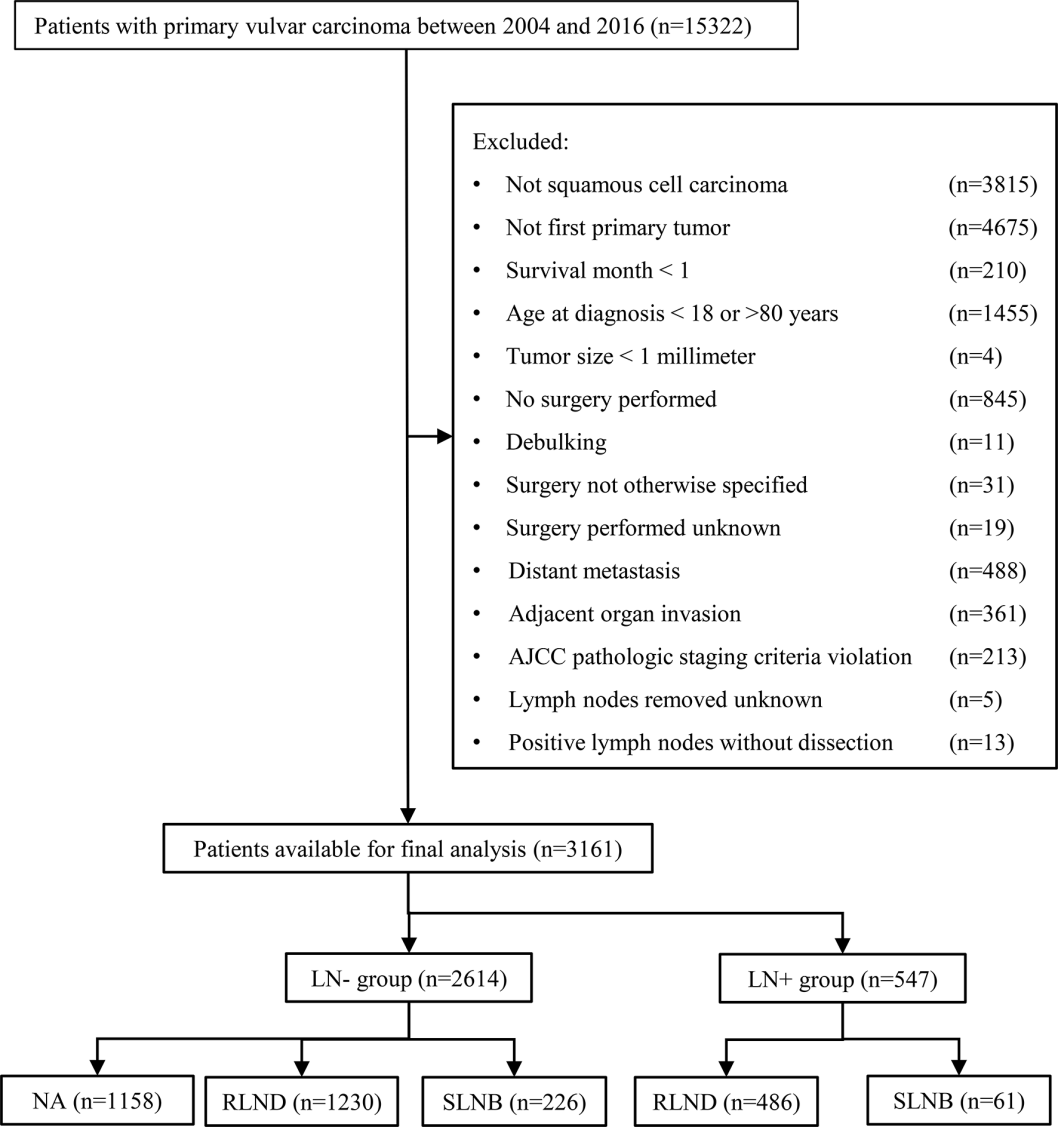
Flowchart of the patient selection procedure. AJCC, American Joint Committee on Cancer; SLNB, sentinel lymph node biopsy; RLND, regional lymph node dissection; NA, no regional lymph node removed.

**Figure 1** shows the sample selection procedure. Of the 3,161 patients in this study, 287 (9.1%) underwent SLNB, 1,716 (54.3%) underwent RLND, and 1,158 (36.6%) had no regional lymph nodes removed. The median [interquartile range, IQR] follow-up of SLNB, RLND, and NA patients were 36 months [20–61 months], 52 months [22–92 months], and 56 months [28–94 months], respectively. The median [IQR] age of SLNB, RLND, and NA patients were 61 [51–69], 59 [50–70], and 58 [49–67], respectively. More SLNB patients were diagnosed after 2010 compared with RLND patients (79.4% *vs*. 54.1%) and have a tumor size of <2 cm (47.0% *vs*. 33.0%) and an invasion depth of >1 mm (77.0% *vs*. 67.2%). Patients who underwent SLNB had a higher percentage of being alive (86.8% *vs*. 70.6%) and negative lymph node findings (78.8% *vs*. 71.7%) compared with those who underwent RLND (**Table 1**).

**Table 1:** Baseline characteristics.

Characteristics	NA	RLND	SLNB
	No. (%)	No. (%)	No. (%)
**Total**	1158	1716	287
**Region**			
East	599 (51.7)	812 (47.3)	96 (33.4)
Northern Plains	115 (9.9)	179 (10.4)	42 (14.6)
Pacific Coast	399 (34.5)	657 (38.3)	130 (45.3)
Southwest	45 (3.9)	68 (4.0)	19 (6.6)
**Insurance status**			
Insured	712 (61.5)	1038 (60.5)	227 (79.1)
Medicaid	145 (12.5)	237 (13.8)	29 (10.1)
Uninsured	36 (3.1)	80 (4.7)	2 (0.7)
Unknown	265 (22.9)	361 (21.0)	29 (10.1)
**Year of diagnosis**			
2004-2009	492 (42.5)	787 (45.9)	59 (20.6)
2010-2016	666 (57.5)	929 (54.1)	228 (79.4)
**Age**			
**median age (IQR), year**	58 (49–67)	59 (50–70)	61 (51–69)
18-49	317 (27.4)	419 (24.4)	60 (20.9)
50-59	326 (28.2)	445 (25.9)	73 (25.4)
60-69	289 (25.0)	410 (23.9)	84 (29.3)
70-80	226 (19.5)	442 (25.8)	70 (24.4)
**Race**			
White	985 (85.1)	1483 (86.4)	268 (93.4)
Black	135 (11.7)	169 (9.8)	9 (3.1)
Other	38 (3.3)	64 (3.7)	10 (3.5)
**Marital status**			
Married	505 (43.6)	777 (45.3)	149 (51.9)
Single	225 (19.4)	350 (20.4)	44 (15.3)
Divorced/separated/widowed	333 (28.8)	507 (29.5)	75 (26.1)
Unknown	95 (8.2)	82 (4.8)	19 (6.6)
**Primary site**			
Labium majus	83 (7.2)	158 (9.2)	23 (8.0)
Labium minus	55 (4.7)	87 (5.1)	19 (6.6)
Clitoris	10 (0.9)	50 (2.9)	5 (1.7)
Overlapping lesion	40 (3.5)	70 (4.1)	6 (2.1)
Vulva, NOS	970 (83.8)	1351 (78.7)	234 (81.5)
**Pathology grade**			
Grade I	373 (32.2)	460 (26.8)	88 (30.7)
Grade II	268 (23.1)	774 (45.1)	131 (45.6)
Grade III/IV	83 (7.2)	323 (18.8)	43 (15.0)
Unknown	434 (37.5)	159 (9.3)	25 (8.7)
**Tumor size, cm**			
<2	627 (54.1)	567 (33.0)	135 (47.0)
2-4	177 (15.3)	577 (33.6)	101 (35.2)
≥4	100 (8.6)	427 (24.9)	21 (7.3)
Unknown	254 (21.9)	145 (8.4)	30 (10.5)
**Invasion depth, mm**			
≤1	496 (42.8)	112 (6.5)	15 (5.2)
>1	278 (24.0)	1153 (67.2)	221 (77.0)
Unknown	384 (33.2)	451 (26.3)	51 (17.8)
**Surgery**			
LTE	387 (33.4)	109 (6.4)	21 (7.3)
SV	585 (50.5)	727 (42.4)	158 (55.1)
TV	87 (7.5)	314 (18.3)	38 (13.2)
RV	99 (8.5)	566 (33.0)	70 (24.4)
**Radiotherapy**			
No	1079 (93.2)	1279 (74.5)	232 (80.8)
Yes	79 (6.8)	437 (25.5)	55 (19.2)
**Chemotherapy**			
No	1125 (97.2)	1519 (88.5)	264 (92.0)
Yes	33 (2.8)	197 (11.5)	23 (8.0)
**Lymph node size, mm**			
<5	1158 (100.0)	1300 (75.8)	242 (84.3)
≥5	–	91 (5.3)	17 (5.9)
Unknown	–	325 (18.9)	28 (9.8)
**Lymph node findings**			
Negative	1158 (100.0)	1230 (71.7)	226 (78.8)
Positive	–	486 (28.3)	61 (21.2)
**Follow-up time (IQR), month**	56 (28–94)	52 (22–92)	36 (20–61)
**Outcome**			
Alive	972 (83.9)	1211 (70.6)	249 (86.8)
Dead from vulvar cancer	67 (5.8)	180 (10.5)	17 (5.9)
Not dead from vulvar cancer	115 (9.9)	316 (18.4)	20 (7.0)
Dead from unknown cause	4 (0.3)	9 (0.5)	1 (0.3)

LN−, negative regional lymph node findings; LN+, positive regional lymph node findings; NA, no regional lymph node removed; SLNB, sentinel lymph node biopsy; RLND, regional lymph node dissection; cm, centimeter; mm, millimeter; IQR, interquartile range.

### Comparison of Overall Survival and Vulvar Cancer-Specific Survival Between the SLNB and RLND Groups

**Figure 2:**
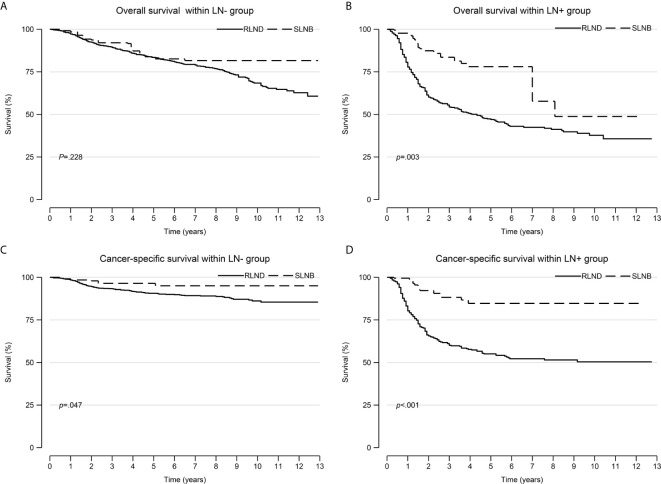
Overall survival and cancer-specific survival curves after inverse probability weighting. **(A)** overall survival within the LN− cohort; **(B)** overall survival within the LN+ cohort; **(C)** cancer-specific survival within the LN− cohort; **(D)** cancer-specific survival within the LN+ cohort. LN−, negative regional lymph node findings; LN+, positive regional lymph node findings.

The IPW adjusted Kaplan-Meier curves of CSS and OS are summarized in **Figure 2**.

**Figure 3:**
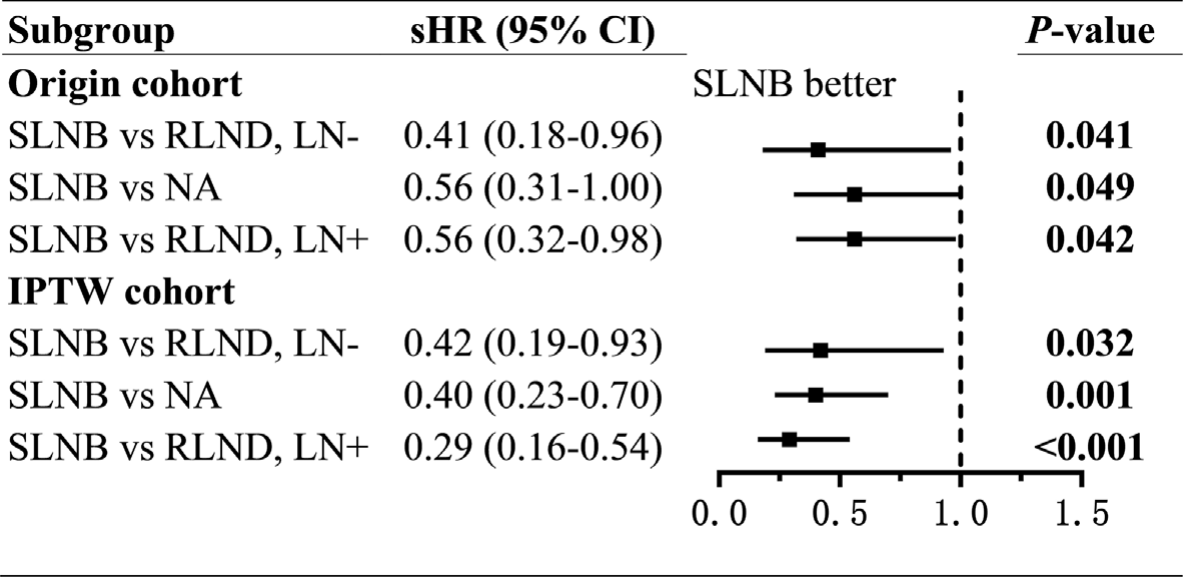
Forest plot of compete-risk subdistribution hazard ratios (sHR) of cancer-specific survival. sHR, subdistribution hazard ratios; SLNB, sentinel lymph node biopsy; RLND, regional lymph node dissection; NA, no regional lymph node removed.

Patients who underwent SLNB had significantly improved CSS than those who underwent RLND both in the LN− cohort (unadjusted sHR=0.41; 95% CI, 0.18–0.96; *P=*0.041; adjusted sHR=0.42, 95% CI 0.19–0.93, *P=*0.032) and in the LN+ cohort (unadjusted sHR=0.56; 95% CI, 0.32–0.98, *P=*0.042; adjusted sHR=0.29; 95% CI, 0.16–0.54, *P<*0.001). Notably, patients who received radiotherapy had worse OS than those who did not (sHR=2.91; 95% CI, 1.32–6.42; *P=*0.008) in the LN− cohort (**Figure 3** and **Supplementary Tables 1**, **2**).

**Figure 4:**
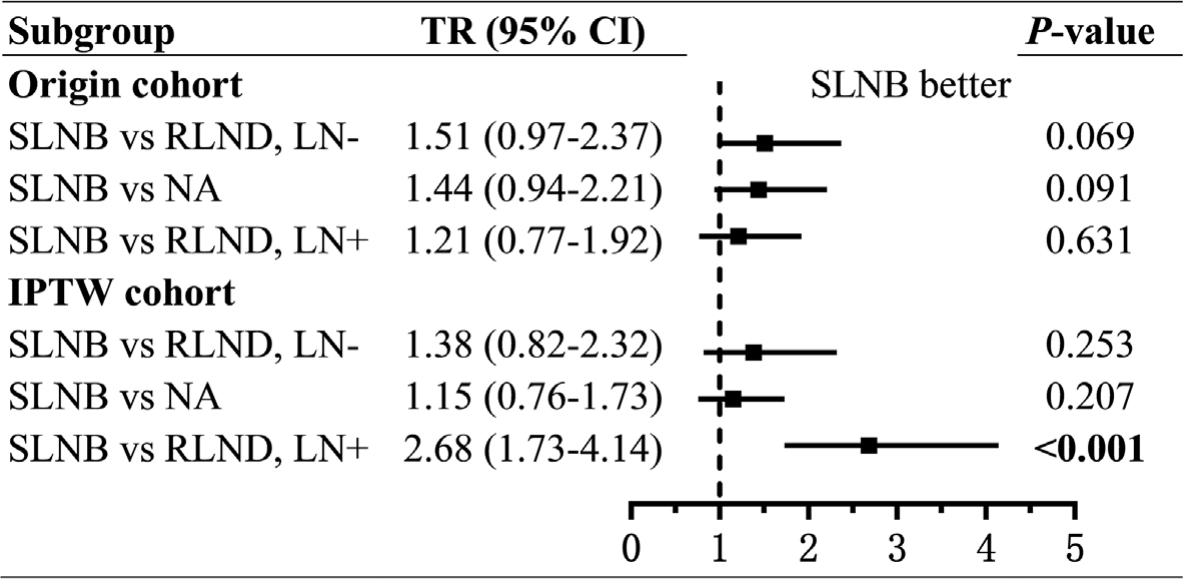
Forest plot of time ratios (TR) of overall survival. TR, time ratio; SLNB, sentinel lymph node biopsy; RLND, regional lymph node dissection; NA, no regional lymph node removed.

As for the OS, patients who underwent SLNB had prolonged OS than those who underwent RLND in the LN− cohort (unadjusted TR=1.51; 95% CI, 0.97–2.37; *P=*0.069; adjusted TR=1.38; 95% CI, 0.82–2.32; *P=*0.226) and in the LN+ cohort (unadjusted TR=1.21; 95% CI, 0.77–1.92; *P=*0.406; adjusted TR=2.68; 95% CI, 1.73–4.14; *P<*0.001), although the effect was not significant in the LN− cohort. (**Figure 4** and **Supplementary Tables 3**, **4**).

Older age was associated with a significant worse CSS (LN−: 18–49, reference; 60-69, adjusted sHR=3.15, *P=*0.005; 70–80, adjusted sHR=8.81, *P*<0.001; LN+: 18–49, reference; 70–80, adjusted sHR=2.19, *P=*0.006) and OS (LN−: 18–49, reference; 60–69, adjusted TR=0.32, *P=*0.002; 70-80, adjusted TR=0.19, *P*<0.001; LN+: 18–49, reference; 7–80, adjusted TR=0.32, *P*<0.001) (**Supplementary Tables 1**-**4**).

### Comparison of Overall Survival and Vulvar Cancer-Specific Survival Between SLNB and NA Groups

To assess the non-inferiority of SLNB compared with NA, we conducted regressions of survival for SLNB and NA groups. Also, we found, compared with patients in the NA group, those who underwent SLNB had significantly prolonged CSS (unadjusted sHR=0.56; 95% CI, 0.31–1.00; *P=*0.049; adjusted sHR = 0.40; 95% CI, 0.23–0.70; *P=*0.001), but similar OS (unadjusted TR=1.44; 95% CI, 0.94–2.21, *P*=0.091; adjusted TR=1.15; 95% CI, 0.76–1.73; *P=*0.279) (**Figures 3**, **4** and **Supplementary Tables 5**, **6**).

### Sensitive Analysis

Because clinical lymph node size was an important factor associated with the choice between SLNB and RLND, we carried out a sensitivity analysis on missing values of lymph node size. We assessed two extreme scenarios: all missing values of lymph node size were considered to be (1) < 5 mm and (2) ≥5 mm. Then, we performed IPW adjusted AFT and CR regressions for OS and CSS within the LN+ cohort and the overall cohort (combination of LN+ and LN−). Finally, the beneficial effect of SLNB on OS and CSS changed slightly but remain consistent (**Supplementary Table 7**).

## Discussion

This study’s key findings were that SLNB led to significantly prolonged survival outcomes in VSCC surgery patients with no distant metastasis and adjacent organ invading compared to RLND and NA. To our knowledge, this study is the first retrospective study comparing SLNB with RLND in VSCC surgery patients who had no distant metastasis and adjacent organ invading, with 287 patients treated with SLNB and a sample size of 3,161 patients. This study controlled for diverse confounding factors, such as surgery type, tumor size, invasion depth, radiotherapy, and positive lymph nodes findings. IPW adjustment was carried out to minimize the imbalance of variables between groups. This study adds to the supportive evidence of the beneficial effect of SLNB on the survival of VSCC surgery patients and extends the application scope of SLNB.

In this study, the beneficial effect of SLNB compared to RLND on CSS was larger in the LN+ cohort(sHR [95% CI]=0.29 [0.16–0.54]) than in the LN− cohort (sHR [95% CI]=0.42 [0.19–0.93]). Similar larger effect of SLNB was also present for OS in the LN+ cohort (TR [95% CI]=2.68 [1.73–4.14]) than in the LN− cohort (TR [95% CI]=1.38 [0.82–2.32]). Thereby, this study demonstrates that patients in the LN+ cohort could benefit more from SLNB than those in the LN− cohort. To explore the non-inferior effect of SLNB, we carried out a comparison between patients treated with SLNB with those with no regional lymph nodes removed, and the result was very promising. Patients treated with SLNB has significantly improved CSS (sHR [95% CI]=0.40 [0.23–0.70]) and similar OS (TR [95% CI]=1.15 [0.76–1.73]) compared with those in the NA group, which indicates that patients in the NA group might have missing detection of microscopic positive lymph nodes. So the extended application of SLNB was feasible.

Because of vulvar carcinoma’s rareness, there have been no random control trials, which may not be feasible because of methodological and ethical issues, providing high-level evidence about the safety and efficacy of SLNB compared with that of RLND (16). This sizable retrospective study confirmed the significantly superior role of SLNB *versus* RLND in VSCC surgery patients within both the LN− and LN+ cohorts. This study found that surgery type, tumor size, and invasion depth did not limit the applying of SLNB, contrary to the previous study’s finding that SLNB should be limited to tumors with size ≤4 cm and invasion depth >1 mm (17). Our study provides evidence for extending the indication of the application of SLNB patients with any tumor size and invasion depth, irrespective of surgery type and regional lymph nodes findings. In contrast, age should be taken into account because of its statistically significant association with survival. Together with the cost-efficacy of SLNB, more VSCC surgery patients will benefit from SLNB (17, 18).

The explanation of the promising survival outcome associate with SLNB may lie in that several innovation techniques of SLNB, including imaging tracer agent (ranging from blue dye to indocyanine green and Technetium-99m colloid albumin as well as their combination) and imaging equipment (from lymphoscintigraphy, single-photon emission computed tomography or computed tomography [SPECT/CT] to a fusion of SPECT/CT and ultrasound), have dramatically progressed the precision of lymph node localization (17, 19, 20). SPECT/CT could currently personalize lymphatic mapping and provide detailed information about the number and anatomical location of sentinel lymph nodes for adequate surgical planning in the groin (21). However, it is still essential to standardize the acquisition principles of SPECT/CT images and centralize SLNB performing in experienced centers for a personalized approach (17).

This study has some limitations. 1) Detailed information about surgery was not available in SEER, for example, hospital’s care quality, imaging equipment, tracer agent for imaging, surgeon’s professional experience. Thus, we could not profoundly control those factors’ impact and handle the heterogeneity of those factors between groups. Moreover, margin status was not reported in the SEER, so it could not be controlled, although margin status was proven to be not a significant prognostic factor of survival in early studies (22–25). 2) This study covered so long a period from 2004 to 2016 that some missing factors may bias the findings, despite the year of diagnosis was grouped into two intervals at 2010 and controlled in IPW calculation and multivariate regression, and no significant survival difference was found to be associated with the year of diagnosis. 3) As the retrospective study’s nature, there might be missing confounders that may be important for analysis, which would lead to bias in our findings. For example, we did not know where the exact location of the tumors. Although we had adjusted for the primary site of the tumor, that might not be adequate to account for the bias caused by tumor location. 4) Despite IPW techniques, residual confounding may exist. 5) The pathological result of SLNB and RLND during the surgery process was unavailable in the SEER database, and only the final pathological histology results were given. Thus the false negatives and false positives that were of great interest could not be calculated.

Although our study had some limitations, it was the first retrospective study investigating SLNB in VSCC surgery patients without distant metastasis and adjacent organ involvement until now. Our study extends the scope of SLNB performing on this rare cancer. Our findings will make clinicians preferentially consider performing SLNB in VSCC surgery patients irrespective of surgery type, invasion depth, and positive lymph node findings so that more patients will benefit from SLNB.

## Conclusions

SLNB results in significantly prolonged survival in VSCC surgery patients without distant metastasis and adjacent organ invading, irrespective of tumor size, surgery type, invasion depth, and positive lymph node findings. SLNB could be carried out preferentially in VSCC surgery patients. Further prospective clinical controlled trials are warranted to confirm the superior efficacy of SLNB.

## Data Availability Statement

Publicly available data sets were analyzed in this study. This data can be found here: https://seer.cancer.gov/.

## Ethics Statement

Ethical review and approval was not required for the study on human participants in accordance with the local legislation and institutional requirements. Written informed consent for participation was not required for this study in accordance with the national legislation and the institutional requirements.

## Author Contributions

WLZ: Conceptualization, Data curation, Formal analyses, Supervision, Writing-Original draft preparation, Writing-Reviewing, and Editing. YB: Conceptualization, Methodology, Software. YYY: Conceptualization, Formal analyses, Methodology, Software, Supervision, Visualization, Writing-Original draft preparation, Writing- Reviewing, and Editing. All authors contributed to the article and approved the submitted version.

## Conflict of Interest

The authors declare that the research was conducted in the absence of any commercial or financial relationships that could be construed as a potential conflict of interest.

## Publisher’s Note

All claims expressed in this article are solely those of the authors and do not necessarily represent those of their affiliated organizations, or those of the publisher, the editors and the reviewers. Any product that may be evaluated in this article, or claim that may be made by its manufacturer, is not guaranteed or endorsed by the publisher.

## Supplementary Material

Supplementary Table 1 Multivariate compete-risk analysis of characteristics associated with cancer-specific survival in the LN− cohort for patients treated with SLNB and RLND. LN−, negative regional lymph node findings; SLNB, sentinel lymph node biopsy; RLND, regional lymph node dissection; IPW, inverse probability weighting; sHR, sub proportional hazard ratio; NOS, not otherwise specified; cm, centimeter; mm, millimeter.

Supplementary Table 2 Multivariate compete-risk analysis of characteristics associated with cancer-specific survival in the LN+ cohort for patients treated with SLNB and RLND. LN+, positive regional lymph node findings; IPW, inverse probability weighting; sHR, sub proportional hazard ratio; NOS, not otherwise specified; SLNB, sentinel lymph node biopsy; RLND, regional lymph node dissection; cm, centimeter; mm, millimeter.

Supplementary Table 3 Multivariate accelerate failure time analysis of characteristics associated with overall survival in the LN− cohort for patients treated with SLNB and RLND. LN−, negative regional lymph node findings; SLNB, sentinel lymph node biopsy; RLND, regional lymph node dissection; IPW, inverse probability weighting; TR, time ratio; NOS, not otherwise specified; cm, centimeter; mm, millimeter.

Supplementary Table 4 Multivariate accelerate failure time analysis of characteristics associated with overall survival in the LN+ cohort for patients treated with SLNB and RLND. LN+, positive regional lymph node findings; SLNB, sentinel lymph node biopsy; RLND, regional lymph node dissection; IPW, inverse probability weighting; TR, time ratio; NOS, not otherwise specified; cm, centimeter; mm, millimeter.

Supplementary Table 5 Multivariate compete-risk analysis of characteristics associated with cancer-specific survival for patients treated with SLNB and NA. LN−, negative regional lymph node findings; SLNB, sentinel lymph node biopsy; NA, no regional lymph node removed; IPW, inverse probability weighting; sHR, sub proportional hazard ratio; NOS, not otherwise specified; cm, centimeter; mm, millimeter.

Supplementary Table 6 Multivariate accelerate failure time analysis of characteristics associated with overall survival for patients treated with SLNB and NA. LN−, negative regional lymph node findings; SLNB, sentinel lymph node biopsy; NA, no regional lymph node removed; IPW, inverse probability weighting; TR, time ratio; NOS, not otherwise specified; cm, centimeter; mm, millimeter.

Supplementary Table 7 Effect of SLNB *versus* RLND from IPW adjusted multivariate AFT and CR models for sensitivity analysis about missing lymph node size. SLNB, sentinel lymph node biopsy; RLND, regional lymph node removed; IPW, inverse probability weighting; AFT, accelerate failure time; CR, compete-risk; TR, time ratio; sHR, sub proportional hazard ratio; LN+, positive regional lymph node findings; LN−, negative regional lymph node findings.

## References

[1] Siegel RL Miller KD Jemal A. Cancer Statistics, 2020. CA Cancer J Clin (2020) 70(1):7–30. 10.3322/caac.21590 31912902

[2] Tan A Bieber AK Stein JA Pomeranz MK. Diagnosis and Management of Vulvar Cancer: A Review. J Am Acad Dermatol (2019) 81(6):1387–96. 10.1016/j.jaad.2019.07.055 31349045

[3] Koh WJ Greer BE Abu-Rustum NR Campos SM Cho KR Chon HS. Vulvar Cancer, Version 1.2017, NCCN Clinical Practice Guidelines in Oncology. J Natl Compr Canc Netw (2017) 15(1):92–120. 10.6004/jnccn.2017.0008 28040721

[4] Dellinger TH Hakim AA Lee SJ Wakabayashi MT Morgan RJ Han ES. Surgical Management of Vulvar Cancer. J Natl Compr Canc Netw (2017) 15(1):121–8. 10.6004/jnccn.2017.0009 28040722

[5] Papadia A Ehm L Gasparri ML Wang J Radan AP Mueller MD. Unilateral *Versus* Bilateral Lymph-Nodal Metastases and Oncologic Outcome in Vulvar Cancer Patients. J Cancer Res Clin Oncol (2020) 146(7):1877–81. 10.1007/s00432-020-03196-9 PMC11804390 32266536

[6] Zhou J Shan G. The Prognostic Role of FIGO Stage in Patients With Vulvar Cancer: A Systematic Review and Meta-Analysis. Curr Med Res Opin (2016) 32(6):1121–30. 10.1185/03007995.2016.1162147 26959073

[7] Hinten F van den Einden LC Hendriks JC van der Zee AG Bulten J Massuger LF. Risk Factors for Short- and Long-Term Complications After Groin Surgery in Vulvar Cancer. Br J Cancer (2011) 105(9):1279–87. 10.1038/bjc.2011.407 PMC3241565 21970884

[8] Pouwer AW Arts HJ van der Velden J de Hullu JA. Limiting the Morbidity of Inguinofemoral Lymphadenectomy in Vulvar Cancer Patients; A Review. Expert Rev Anticancer Ther (2017) 17(7):615–24. 10.1080/14737140.2017.1337513 28608762

[9] Levenback CF Ali S Coleman RL Gold MA Fowler JM Judson PL. Lymphatic Mapping and Sentinel Lymph Node Biopsy in Women With Squamous Cell Carcinoma of the Vulva: A Gynecologic Oncology Group Study. J Clin Oncol (2012) 30(31):3786–91. 10.1200/jco.2011.41.2528 PMC3478573 22753905

[10] Meads C Sutton A Małysiak S Kowalska M Zapalska A Rogozinska E. Sentinel Lymph Node Status in Vulval Cancer: Systematic Reviews of Test Accuracy and Decision-Analytic Model-Based Economic Evaluation. Health Technol Assess (2013) 17(60):1–216. 10.3310/hta17600 PMC4781286 24331128

[11] Van Dyne EA Henley SJ Saraiya M Thomas CC Markowitz LE Benard VB. Trends in Human Papillomavirus-Associated Cancers - United States, 1999-2015. MMWR Morbidity Mortality Weekly Rep (2018) 67(33):918–24. 10.15585/mmwr.mm6733a2 PMC6107321 30138307

[12] Thaker NG Klopp AH Jhingran A Frumovitz M Iyer RB Eifel PJ. Survival Outcomes for Patients With Stage IVB Vulvar Cancer With Grossly Positive Pelvic Lymph Nodes: Time to Reconsider the FIGO Staging System? Gynecol Oncol (2015) 136(2):269–73. 10.1016/j.ygyno.2014.12.013 PMC4329262 25524458

[13] Höckel M Trott S Dornhöfer N Horn LC Hentschel B Wolf B. Vulvar Field Resection Based on Ontogenetic Cancer Field Theory for Surgical Treatment of Vulvar Carcinoma: A Single-Centre, Single-Group, Prospective Trial. Lancet Oncol (2018) 19(4):537–48. 10.1016/s1470-2045(18)30109-8 29530664

[14] Alhatem A Lambert WC Karanfilian K Behbahani S Heller D. Impact of Partnership Status on Clinical Outcomes of Patients With Vulvar Squamous Cell Carcinoma and Performance of Sentinel Lymph Node Biopsy. Int J Gynecol Cancer (2020) 30(5):583–9. 10.1136/ijgc-2019-001001 32184269

[15] Shinde A Li R Amini A Chen YJ Cristea M Wang W. Role of Locoregional Treatment in Vulvar Cancer With Pelvic Lymph Node Metastases: Time to Reconsider Figo Staging? J Natl Compr Canc Netw (2019) 17(8):922–30. 10.6004/jnccn.2019.7288 31390593

[16] Lawrie TA Patel A Martin-Hirsch PP Bryant A Ratnavelu ND Naik R. Sentinel Node Assessment for Diagnosis of Groin Lymph Node Involvement in Vulval Cancer. Cochrane Database Syst Rev (2014) 2014(6):Cd010409. 10.1002/14651858.CD010409.pub2 PMC6457826 24970683

[17] Collarino A Fuoco V Garganese G Pereira Arias-Bouda LM Perotti G Manca G. Lymphoscintigraphy and Sentinel Lymph Node Biopsy in Vulvar Carcinoma: Update From a European Expert Panel. Eur J Nucl Med Mol Imaging (2020) 47(5):1261–74. 10.1007/s00259-019-04650-8 31897584

[18] Sutton AJ Barton P Sundar S Meads C Rosenthal AN Baldwin P. Cost-Effectiveness of Sentinel Lymph Node Biopsy *vs* Inguinofemoral Lymphadenectomy in Women With Vulval Cancer. Br J Cancer (2013) 109(10):2533–47. 10.1038/bjc.2013.631 PMC3833218 24129233

[19] Schaafsma BE Verbeek FP Peters AA van der Vorst JR de Kroon CD van Poelgeest MI. Near-Infrared Fluorescence Sentinel Lymph Node Biopsy in Vulvar Cancer: A Randomised Comparison of Lymphatic Tracers. BJOG (2013) 120(6):758–64. 10.1111/1471-0528.12173 PMC3622799 23418877

[20] Zekan J Mutvar A Huic D Petrovic D Karelovic D Mitrovic L. Reliability of Sentinel Node Assay in Vulvar Cancer: The First Croatian Validation Trial. Gynecol Oncol (2012) 126(1):99–102. 10.1016/j.ygyno.2012.04.001 22503824

[21] Collarino A Donswijk ML van Driel WJ Stokkel MP Valdés Olmos RA. The Use of SPECT/CT for Anatomical Mapping of Lymphatic Drainage in Vulvar Cancer: Possible Implications for the Extent of Inguinal Lymph Node Dissection. Eur J Nucl Med Mol Imaging (2015) 42(13):2064–71. 10.1007/s00259-015-3127-1 26219869

[22] Te Grootenhuis NC Pouwer AW de Bock GH Hollema H Bulten J van der Zee AGJ. Margin Status Revisited in Vulvar Squamous Cell Carcinoma. Gynecol Oncol (2019) 154(2):266–75. 10.1016/j.ygyno.2019.05.010 31109660

[23] Raimond E Delorme C Ouldamer L Carcopino X Bendifallah S Touboul C. Surgical Treatment of Vulvar Cancer: Impact of Tumor-Free Margin Distance on Recurrence and Survival. A Multicentre Cohort Analysis From the Francogyn Study Group. Eur J Surg Oncol (2019) 45(11):2109–14. 10.1016/j.ejso.2019.07.005 31285094

[24] Nooij LS van der Slot MA Dekkers OM Stijnen T Gaarenstroom KN Creutzberg CL. Tumour-Free Margins in Vulvar Squamous Cell Carcinoma: Does Distance Really Matter? Eur J Cancer (2016) 65:139–49. 10.1016/j.ejca.2016.07.006 27497345

[25] Woelber L Griebel LF Eulenburg C Sehouli J Jueckstock J Hilpert F. Role of Tumour-Free Margin Distance for Loco-Regional Control in Vulvar Cancer-a Subset Analysis of the Arbeitsgemeinschaft Gynäkologische Onkologie CaRE-1 Multicenter Study. Eur J Cancer (2016) 69:180–8. 10.1016/j.ejca.2016.09.038 27837710

