# Supplementary material for: Safety and Benefit Of Sentinel Lymph Nodes Biopsy Compared to Regional Lymph Node Dissection in Primary Vulvar Cancer Patients Without Distant Metastasis and Adjacent Organ Invasion: A Retrospective Population Study
Source: Front Oncol. 2021 Jul 26;11:676038. doi: 10.3389/fonc.2021.676038 (PMC8350928; doi:10.3389/fonc.2021.676038)
Supplement: Supplementary Table 1 — Multivariate compete-risk analysis of characteristics associated with cancer-specific survival in the LN− cohort for patients treated with SLNB and RLND. LN−, negative regional lymph node findings; SLNB, sentinel lymph node biopsy; RLND, regional lymph node dissection; IPW, inverse probability weighting; sHR, sub proportional hazard ratio; NOS, not otherwise specified; cm, centimeter; mm, millimeter. [file Table_1.docx]

**Supplementary Table 1 |** **Multivariate compete-risk model of characteristics associated with cancer-specific survival in the LN- cohort for patients treated with SLNB and RLND**

| **Characteristics** | **Origin cohort** | | **IPW cohort** | |
| --- | --- | --- | --- | --- |
|  | Unadjusted sHR(95%CI) | *P* | Adjusted sHR(95%CI) | *P* |
| **Region** |  |  |  |  |
| East | Reference |  | Reference |  |
| Northern Plains | 1.41 (0.80-2.47) | 0.235 | 0.95 (0.45-2.04) | 0.903 |
| Pacific Coast | 1.13 (0.75-1.72) | 0.554 | 0.66 (0.37-1.18) | 0.162 |
| Southwest | 0.62 (0.21-1.88) | 0.400 | 0.37 (0.11-1.21) | 0.100 |
| **Insurance** |  |  |  |  |
| Insured | Reference |  | Reference |  |
| Medicaid | 1.33 (0.73-2.41) | 0.347 | 0.85 (0.41-1.76) | 0.664 |
| Uninsured | 0.90 (0.20-4.06) | 0.896 | 0.56 (0.11-2.98) | 0.503 |
| Unknown | 1.46 (0.91-2.36) | 0.120 | 0.90 (0.47-1.73) | 0.747 |
| **Year of diagnosis** |  |  |  |  |
| 2004-2009 | Reference |  | Reference |  |
| 2010-2016 | 1.03 (0.64-1.67) | 0.899 | 0.91 (0.51-1.61) | 0.741 |
| **Age, year** |  |  |  |  |
| 18-49 | Reference |  | Reference |  |
| 50-59 | 1.54 (0.72-3.31) | 0.268 | 1.59 (0.70-3.59) | 0.268 |
| 60-69 | 2.78 (1.35-5.71) | **0.006** | 3.15 (1.42-6.96) | **0.005** |
| 70-80 | 5.95 (3.01-11.76) | **<0.001** | 8.81 (4.33-17.94) | **<0.001** |
| **Race** |  |  |  |  |
| White | Reference |  | Reference |  |
| Black | 0.50 (0.20-1.30) | 0.156 | 0.33 (0.11-0.99) | 0.047 |
| Other | 1.59 (0.72-3.54) | 0.252 | 1.28 (0.55-3.01) | 0.566 |
| **Marital status** |  |  |  |  |
| Married | Reference |  | Reference |  |
| Single | 0.71 (0.40-1.26) | 0.243 | 0.55 (0.26-1.17) | 0.122 |
| Divorced/separated/widowed | 0.78 (0.51-1.21) | 0.274 | 0.55 (0.33-0.94) | **0.027** |
| Unknown | 0.31 (0.07-1.32) | 0.113 | 0.22 (0.05-1.00) | **0.049** |
| **Primary site** |  |  |  |  |
| Labium majus | Reference |  | Reference |  |
| Labium minus | 1.88 (0.71-4.97) | 0.202 | 1.45 (0.42-4.92) | 0.555 |
| Clitoris | 0.99 (0.26-3.73) | 0.986 | 1.11 (0.27-4.51) | 0.882 |
| Overlapping lesion | 1.02 (0.27-3.82) | 0.980 | 0.86 (0.20-3.68) | 0.841 |
| Vulva, NOS | 1.58 (0.75-3.32) | 0.227 | 1.81 (0.76-4.32) | 0.183 |
| **Pathology grade** |  |  |  |  |
| Grade I | Reference |  | Reference |  |
| Grade II | 1.25 (0.80-1.96) | 0.324 | 1.51 (0.84-2.70) | 0.166 |
| Grade III/IV | 1.98 (1.16-3.37) | **0.012** | 3.38 (1.72-6.62) | **<0.001** |
| Unknown | 0.50 (0.20-1.28) | 0.147 | 0.50 (0.19-1.30) | 0.156 |
| **Tumor size, cm** |  |  |  |  |
| <2 | Reference |  | Reference |  |
| 2-4 | 1.45 (0.93-2.25) | 0.100 | 1.47 (0.87-2.48) | 0.153 |
| ≥4 | 1.32 (0.75-2.32) | 0.344 | 1.52 (0.63-3.63) | 0.349 |
| Unknown | 0.82 (0.38-1.77) | 0.610 | 1.25 (0.40-3.87) | 0.697 |
| **Invasion depth, mm** |  |  |  |  |
| ≤1 | Reference |  | Reference |  |
| >1 | 0.71 (0.30-1.69) | 0.444 | 0.85 (0.35-2.07) | 0.719 |
| Unknown | 0.95 (0.40-2.26) | 0.900 | 0.86 (0.35-2.12) | 0.739 |
| **Surgery** |  |  |  |  |
| LTE | Reference |  | Reference |  |
| SV | 1.48 (0.55-3.97) | 0.433 | 1.77 (0.62-5.07) | 0.289 |
| TV | 1.93 (0.68-5.47) | 0.218 | 2.55 (0.81-8.00) | 0.108 |
| RV | 1.71 (0.62-4.66) | 0.297 | 1.21 (0.40-3.69) | 0.738 |
| **Radiotherapy** |  |  |  |  |
| No | Reference |  | Reference |  |
| Yes | 2.36 (1.30-4.29) | **0.005** | 2.91 (1.32-6.42) | **0.008** |
| **Treatment** |  |  |  |  |
| RLND | Reference |  | Reference |  |
| SLNB | 0.41 (0.18-0.96) | **0.041** | 0.42 (0.19-0.93) | **0.032** |

*Abbreviations: LN-, negative regional lymph node findings; SLNB, sentinel lymph node biopsy; RLND, regional lymph node dissection; IPW, inverse probability weighting; sHR, sub proportional hazard ratio; NOS, not otherwise specified; cm, centimeter; mm, millimeter*
