# Supplementary material for: Safety and Benefit Of Sentinel Lymph Nodes Biopsy Compared to Regional Lymph Node Dissection in Primary Vulvar Cancer Patients Without Distant Metastasis and Adjacent Organ Invasion: A Retrospective Population Study
Source: Front Oncol. 2021 Jul 26;11:676038. doi: 10.3389/fonc.2021.676038 (PMC8350928; doi:10.3389/fonc.2021.676038)
Supplement: Supplementary Table 2 — Multivariate compete-risk analysis of characteristics associated with cancer-specific survival in the LN+ cohort for patients treated with SLNB and RLND. LN+, positive regional lymph node findings; IPW, inverse probability weighting; sHR, sub proportional hazard ratio; NOS, not otherwise specified; SLNB, sentinel lymph node biopsy; RLND, regional lymph node dissection; cm, centimeter; mm, millimeter. [file Table_2.docx]

**Supplementary Table 2 | Multivariate compete-risk model of characteristics associated with cancer-specific survival in the LN+ cohort for patients treated with SLNB and RLND**

| **Characteristics** | **Origin cohort** | | **IPW cohort** | |
| --- | --- | --- | --- | --- |
|  | Unadjusted  sHR(95%CI) | *P* | Adjusted  sHR(95%CI) | *P* |
| **Region** |  |  |  |  |
| East | Reference |  | Reference |  |
| Northern Plains | 1.06 (0.66-1.72) | 0.797 | 0.88 (0.50-1.56) | 0.673 |
| Pacific Coast | 1.01 (0.74-1.38) | 0.964 | 1.20 (0.79-1.82) | 0.399 |
| Southwest | 1.84 (0.89-3.80) | 0.102 | 0.86 (0.32-2.30) | 0.762 |
| **Insurance status** |  |  |  |  |
| Insured | Reference |  | Reference |  |
| Medicaid | 1.24 (0.81-1.89) | 0.322 | 1.17 (0.68-2.01) | 0.562 |
| Uninsured | 1.16 (0.47-2.90) | 0.744 | - | - |
| Unknown | 1.30 (0.84-2.00) | 0.238 | 1.83 (1.07-3.12) | **0.027** |
| **Year of diagnosis** |  |  |  |  |
| 2004-2009 | Reference |  | Reference |  |
| 2010-2016 | 1.38 (0.91-2.09) | 0.128 | 1.64 (0.87-2.11) | 0.063 |
| **Age, year** |  |  |  |  |
| 18-49 | Reference |  | Reference |  |
| 50-59 | 0.80 (0.47-1.34) | 0.392 | 0.76 (0.37-1.56) | 0.449 |
| 60-69 | 1.64 (1.01-2.65) | 0.045 | 1.34 (0.72-2.36) | 0.376 |
| 70-80 | 1.99 (1.27-3.12) | **0.003** | 2.19 (1.26-3.81) | **0.006** |
| **Race** |  |  |  |  |
| White | Reference |  | Reference |  |
| Black | 0.51 (0.25-1.02) | 0.058 | 0.21 (0.07-0.64) | **0.006** |
| Other | 0.79 (0.37-1.68) | 0.540 | 0.54 (0.23-1.27) | 0.157 |
| **Marital status** |  |  |  |  |
| Married | Reference |  | Reference |  |
| Single | 1.20 (0.80-1.81) | 0.373 | 1.09 (0.69-1.84) | 0.743 |
| Divorced/separated/widowed | 1.00 (0.71-1.42) | 0.984 | 0.96 (0.62-1.50) | 0.855 |
| Unknown | 1.19 (0.61-2.34) | 0.611 | 1.79 (0.76-4.22) | 0.181 |
| **Primary site** |  |  |  |  |
| Labium majus | Reference |  | Reference |  |
| Labium minus | 0.56 (0.19-1.66) | 0.293 | 0.42 (0.12-1.46) | 0.175 |
| Clitoris | 0.64 (0.24-1.71) | 0.378 | 0.33 (0.07-1.49) | 0.149 |
| Overlapping lesion | 1.57 (0.73-3.36) | 0.246 | 1.91 (0.75-4.87) | 0.173 |
| Vulva, NOS | 1.57 (0.95-2.57) | 0.076 | 1.62 (0.79-3.36) | 0.190 |
| **Pathology grade** |  |  |  |  |
| Grade I | Reference |  | Reference |  |
| Grade II | 1.33 (0.86-2.04) | 0.198 | 1.47 (0.84-2.58) | 0.182 |
| Grade III/IV | 1.22 (0.77-1.93) | 0.402 | 0.90 (0.50-1.62) | 0.719 |
| Unknown | 1.22 (0.45-3.32) | 0.703 | 0.99 (0.31-3.18) | 0.984 |
| **Tumor size, cm** |  |  |  |  |
| <2 | Reference |  | Reference |  |
| 2-4 | 1.30 (0.83-2.05) | 0.253 | 1.40 (0.74-2.62) | 0.301 |
| ≥4 | 2.14 (1.37-3.34) | **0.002** | 1.42 (0.77-2.62) | 0.267 |
| Unknown | 0.24 (0.03-2.30) | 0.217 | 0.18 (0.02-1.61) | 0.126 |
| **Invasion depth，mm** |  |  |  |  |
| ≤1 | Reference |  | Reference |  |
| ＞1 | 0.63 (0.35-1.12) | 0.113 | 0.53 (0.25-1.15) | 0.108 |
| Unknown | 0.67 (0.36-1.23) | 0.197 | 0.68 (0.29-1.57) | 0.364 |
| **Surgery** |  |  |  |  |
| LTE | Reference |  | Reference |  |
| SV | 0.79 (0.35-1.77) | 0.564 | 1.10 (0.45-2.68) | 0.833 |
| TV | 0.75 (0.32-1.76) | 0.511 | 0.99 (0.39-2.47) | 0.979 |
| RV | 0.96 (0.43-2.17) | 0.930 | 1.32 (0.55-3.14) | 0.533 |
| **Radiotherapy** |  |  |  |  |
| No | Reference |  | Reference |  |
| Yes | 1.34 (0.95-1.89) | 0.092 | 1.12 (0.74-1.72) | 0.590 |
| **Lymph node size, mm** |  |  |  |  |
| **<5** | Reference |  | Reference |  |
| **≥5** | 1.06 (0.60-1.85) | 0.845 | 1.54 (0.79-2.98) | 0.204 |
| **Unknown** | 1.96 (1.20-3.18) | 0.007 | 1.78 (0.98-3.23) | 0.057 |
| **Treatment** |  |  |  |  |
| RLND | Reference |  | Reference |  |
| SLNB | 0.56 (0.32-0.98) | 0.042 | 0.29 (0.16-0.54) | **<0.001** |

*Abbreviations: LN+, positive regional lymph node findings;* *SLNB, sentinel lymph node biopsy; RLND, regional lymph node dissection; IPW, inverse probability weighting; sHR, sub proportional hazard ratio; NOS, not otherwise specified; cm, centimeter; mm, millimeter*
