# Supplementary material for: Safety and Benefit Of Sentinel Lymph Nodes Biopsy Compared to Regional Lymph Node Dissection in Primary Vulvar Cancer Patients Without Distant Metastasis and Adjacent Organ Invasion: A Retrospective Population Study
Source: Front Oncol. 2021 Jul 26;11:676038. doi: 10.3389/fonc.2021.676038 (PMC8350928; doi:10.3389/fonc.2021.676038)
Supplement: Supplementary Table 3 — Multivariate accelerate failure time analysis of characteristics associated with overall survival in the LN− cohort for patients treated with SLNB and RLND. LN−, negative regional lymph node findings; SLNB, sentinel lymph node biopsy; RLND, regional lymph node dissection; IPW, inverse probability weighting; TR, time ratio; NOS, not otherwise specified; cm, centimeter; mm, millimeter. [file Table_3.docx]

**Supplementary Table 3 | Multivariate accelerate failure time model of characteristics associated with overall survival in the LN- cohort for patients treated with SLNB and RLND**

| **Characteristics** | **Origin cohort** | | **IPW cohort** | |
| --- | --- | --- | --- | --- |
|  | Unadjusted  TR(95%CI) | *P* | Adjusted  TR(95%CI) | *P* |
| **Region** |  |  |  |  |
| East | Reference |  | Reference |  |
| Northern Plains | 0.79 (0.55-1.14) | 0.210 | 0.60 (0.32-1.13) | 0.115 |
| Pacific Coast | 1.01 (0.78-1.31) | 0.950 | 1.13 (0.74-1.71) | 0.574 |
| Southwest | 1.28 (0.70-2.34) | 0.428 | 1.42 (0.67-3.01) | 0.354 |
| **Insurance** |  |  |  |  |
| Insured | Reference |  | Reference |  |
| Medicaid | 0.71 (0.49-1.03) | 0.070 | 1.27 (0.77-2.11) | 0.348 |
| Uninsured | 0.87 (0.39-1.94) | 0.738 | 2.03 (0.80-5.16) | 0.138 |
| Unknown | 0.85 (0.62-1.15) | 0.288 | 1.13 (0.66-1.95) | 0.652 |
| **Year of diagnosis** |  |  |  |  |
| 2004-2009 | Reference |  | Reference |  |
| 2010-2016 | 1.11 (0.83-1.48) | 0.499 | 1.54 (1.04-2.28) | **0.030** |
| **Age, year** |  |  |  |  |
| 18-49 | Reference |  | Reference |  |
| 50-59 | 0.60 (0.39-0.92) | **0.018** | 0.54 (0.29-1.01) | **0.054** |
| 60-69 | 0.34 (0.23-0.51) | **<0.001** | 0.32 (0.16-0.66) | **0.002** |
| 70-80 | 0.18 (0.12-0.26) | **<0.001** | 0.19 (0.10-0.36) | **<0.001** |
| **Race** |  |  |  |  |
| White | Reference |  | Reference |  |
| Black | 0.90 (0.59-1.37) | 0.629 | 0.51 (0.23-1.11) | 0.088 |
| Other | 0.80 (0.45-1.44) | 0.459 | 0.89 (0.48-1.66) | 0.719 |
| **Marital status** |  |  |  |  |
| Married | Reference |  | Reference |  |
| Single | 1.04 (0.73-1.49) | 0.811 | 0.93 (0.44-1.78) | 0.821 |
| Divorced/separated/widowed | 0.77 (0.59-1.00) | 0.053 | 0.73 (0.48-1.09) | 0.123 |
| Unknown | 1.40 (0.67-2.92) | 0.374 | 1.86 (0.83-417) | 0.132 |
| **Primary site** |  |  |  |  |
| Labium majus | Reference |  | Reference |  |
| Labium minus | 0.85 (0.46-1.59) | 0.620 | 1.11 (0.51-2.43) | 0.785 |
| Clitoris | 0.62 (0.29-1.29) | 0.198 | 0.38 (0.14-1.00) | **0.049** |
| Overlapping lesion | 1.00 (0.46-2.19) | 0.992 | 0.72 (0.28-1.88) | 0.502 |
| Vulva, NOS | 0.83 (0.54-1.28) | 0.399 | 0.66 (0.37-1.15) | 0.142 |
| **Pathology grade** |  |  |  |  |
| Grade I | Reference |  | Reference |  |
| Grade II | 0.92 (0.70-1.21) | 0.555 | 0.74 (0.51-1.09) | 0.127 |
| Grade III/IV | 0.73 (0.50-1.08) | 0.116 | 0.57 (0.34-0.96) | **0.034** |
| Unknown | 1.25 (0.83-1.90) | 0.289 | 0.91 (0.43-1.91) | 0.799 |
| **Tumor size, cm** |  |  |  |  |
| <2 | Reference |  | Reference |  |
| 2-4 | 0.76 (0.57-1.02) | 0.067 | 0.80 (0.51-1.21) | 0.316 |
| ≥4 | 0.68 (0.48-0.95) | **0.022** | 0.55 (0.31-0.99) | **0.048** |
| Unknown | 1.22 (0.76-1.96) | 0.401 | 1.68 (0.88-3.19) | 0.116 |
| **Invasion depth, mm** |  |  |  |  |
| ≤1 | Reference |  | Reference |  |
| >1 | 0.95 (0.59-1.54) | 0.833 | 1.17 (0.64-2.16) | 0.611 |
| Unknown | 0.82 (0.49-1.36) | 0.437 | 1.16 (0.58-2.31) | 0.673 |
| **Surgery** |  |  |  |  |
| LTE | Reference |  | Reference |  |
| SV | 0.63 (0.38-1.07) | 0.085 | 0.59 (0.28-1.25) | 0.170 |
| TV | 0.54 (0.30-0.97) | 0.038 | 0.54 (0.24-1.20) | 0.130 |
| RV | 0.57 (0.34-0.98) | 0.040 | 0.76 (0.38-1.55) | 0.457 |
| **Radiotherapy** |  |  |  |  |
| No | Reference |  | Reference |  |
| Yes | 0.66 (0.43-1.01) | 0.055 | 0.73 (0.39-1.39) | 0.345 |
| **Treatment** |  |  |  |  |
| RLND | Reference |  | Reference |  |
| SLNB | 1.51 (0.97-2.37) | 0.069 | 1.38 (0.82-2.32) | 0.226 |

*Abbreviations: LN-, negative regional lymph node findings; SLNB, sentinel lymph node biopsy; RLND, regional lymph node dissection; IPW, inverse probability weighting; TR, time ratio; NOS, not otherwise specified; cm, centimeter; mm, millimeter*
