# Supplementary material for: Safety and Benefit Of Sentinel Lymph Nodes Biopsy Compared to Regional Lymph Node Dissection in Primary Vulvar Cancer Patients Without Distant Metastasis and Adjacent Organ Invasion: A Retrospective Population Study
Source: Front Oncol. 2021 Jul 26;11:676038. doi: 10.3389/fonc.2021.676038 (PMC8350928; doi:10.3389/fonc.2021.676038)
Supplement: Supplementary Table 4 — Multivariate accelerate failure time analysis of characteristics associated with overall survival in the LN+ cohort for patients treated with SLNB and RLND. LN+, positive regional lymph node findings; SLNB, sentinel lymph node biopsy; RLND, regional lymph node dissection; IPW, inverse probability weighting; TR, time ratio; NOS, not otherwise specified; cm, centimeter; mm, millimeter. [file Table_4.docx]

**Supplementary Table 4 | Multivariate accelerate failure time model of characteristics associated with overall survival in the LN+ cohort for patients treated with SLNB and RLND**

| **Characteristics** | **Origin cohort** | | **IPW cohort** | |
| --- | --- | --- | --- | --- |
|  | Unadjusted  TR(95%CI) | *P* | Adjusted  TR(95%CI) | *P* |
| **Region** |  |  |  |  |
| East | Reference |  | Reference |  |
| Northern Plains | 0.97 (0.63-1.49) | 0.729 | 1.34 (0.80-2.24) | 0.271 |
| Pacific Coast | 1.28 (0.97-1.69) | 0.066 | 1.36 (0.92-2.01) | 0.127 |
| Southwest | 0.61 (0.32-1.15) | 0.141 | 0.57 (0.27-1.19) | 0.135 |
| **Insurance** |  |  |  |  |
| Insured | Reference |  | Reference |  |
| Medicaid | 0.94 (0.63-1.41) | 0.777 | 0.8 1(0.49-1.36) | 0.435 |
| Uninsured | 0.97 (0.45-2.07) | 0.931 | - | - |
| Unknown | 0.77 (0.53-1.14) | 0.196 | 0.62 (0.39-0.99) | **0.048** |
| **Year of diagnosis** |  |  |  |  |
| 2004-2009 | Reference |  | Reference |  |
| 2010-2016 | 0.57 (0.40-0.82) | **0.003** | 0.66 (0.43-1.03) | 0.067 |
| **Age, year** |  |  |  |  |
| 18-49 | Reference |  | Reference |  |
| 50-59 | 1.13 (0.71-1.80) | 0.618 | 1.14 (0.65-1.98) | 0.651 |
| 60-69 | 0.49 (0.31-0.75) | **0.001** | 0.62 (0.37-1.02) | **0.059** |
| 70-80 | 0.37 (0.24-0.56) | **<0.001** | 0.35 (0.22-0.56) | **<0.001** |
| **Race** |  |  |  |  |
| White | Reference |  | Reference |  |
| Black | 1.46 (0.82-2.60) | 0.196 | 2.09 (0.97-4.50) | 0.060 |
| Other | 1.37 (0.70-2.69) | 0.363 | 1.41 (0.75-2.62) | 0.284 |
| **Marital status** |  |  |  |  |
| Married | Reference |  | Reference |  |
| Single | 0.77 (0.53-1.11) | 0.164 | 0.69 (0.42-1.12) | 0.131 |
| Divorced/separated/widowed | 0.89 (0.66-1.21) | 0.460 | 0.90 (0.60-1.36) | 0.628 |
| Unknown | 0.69 (0.41-1.15) | 0.151 | 0.38 (0.19-0.77) | 0.007 |
| **Primary site** |  |  |  |  |
| Labium majus | Reference |  | Reference |  |
| Labium minus | 0.91 (0.38-2.20) | 0.841 | 1.21 (0.45-3.25) | 0.706 |
| Clitoris | 1.50 (0.61-3.65) | 0.374 | 2.98 (1.11-7.98) | **0.030** |
| Overlapping lesion | 0.70 (0.37-1.34) | 0.285 | 0.34 (0.15-0.80) | **0.013** |
| Vulva, NOS | 0.70 (0.44-1.10) | 0.124 | 0.74 (0.44-1.24) | 0.248 |
| **Pathology grade** |  |  |  |  |
| Grade I | Reference |  | Reference |  |
| Grade II | 0.97 (0.65-1.45) | 0.888 | 0.98 (0.51-1.88) | 0.948 |
| Grade III/IV | 1.14 (0.75-1.74) | 0.547 | 1.41 (0.73-2.70) | 0.307 |
| Unknown | 0.66 (0.29-1.49) | 0.318 | 0.69 (0.27-1.77) | 0.444 |
| **Tumor size, cm** |  |  |  |  |
| <2 | Reference |  | Reference |  |
| 2-4 | 0.82 (0.55-1.22) | 0.327 | 0.68 (0.39-1.19) | 0.176 |
| ≥4 | 0.44 (0.30-0.67) | **<0.001** | 0.88 (0.50-1.57) | 0.670 |
| Unknown | 2.47 (0.62-9.79) | 0.197 | 3.88 (1.07-14.08) | **0.040** |
| **Invasion depth, mm** |  |  |  |  |
| ≤1 | Reference |  | Reference |  |
| >1 | 0.95 (0.51-1.78) | 0.882 | 1.29 (0.54-3.09) | 0.570 |
| Unknown | 1.09 (0.57-2.08) | 0.806 | 1.19 (0.46-3.06) | 0.716 |
| **Surgery** |  |  |  |  |
| LTE | Reference |  | Reference |  |
| SV | 1.62 (0.88-2.99) | 0.122 | 0.98 (0.40-2.38) | 0.958 |
| TV | 1.74 (0.90-3.36) | 0.101 | 1.11 (0.44-2.83) | 0.822 |
| RV | 1.13 (0.61-2.10) | 0.686 | 0.51 (0.21-1.26) | 0.145 |
| **Radiotherapy** |  |  |  |  |
| No | Reference |  | Reference |  |
| Yes | 0.92 (0.69-1.22) | 0.558 | 1.18 (0.81-1.72) | 0.383 |
| **Lymph node size, mm** |  |  |  |  |
| **<5** | Reference |  | Reference |  |
| **≥5** | 0.99 (0.62-1.59) | 0.965 | 0.66 (0.36-1.19) | 0.165 |
| **Unknown** | 0.46 (0.30-0.70) | **<0.001** | 0.54 (0.32-0.89) | **0.016** |
| **Treatment** |  |  |  |  |
| RLND | Reference |  | Reference |  |
| SLNB | 1.21 (0.77-1.92) | 0.406 | 2.68 (1.73-4.14) | **<0.001** |

*Abbreviations: LN+, positive regional lymph node findings; SLNB, sentinel lymph node biopsy; RLND, regional lymph node dissection; IPW, inverse probability weighting; TR, time ratio; NOS, not otherwise specified; cm, centimeter; mm, millimeter*
