# Supplementary material for: Safety and Benefit Of Sentinel Lymph Nodes Biopsy Compared to Regional Lymph Node Dissection in Primary Vulvar Cancer Patients Without Distant Metastasis and Adjacent Organ Invasion: A Retrospective Population Study
Source: Front Oncol. 2021 Jul 26;11:676038. doi: 10.3389/fonc.2021.676038 (PMC8350928; doi:10.3389/fonc.2021.676038)
Supplement: Supplementary Table 5 — Multivariate compete-risk analysis of characteristics associated with cancer-specific survival for patients treated with SLNB and NA. LN−, negative regional lymph node findings; SLNB, sentinel lymph node biopsy; NA, no regional lymph node removed; IPW, inverse probability weighting; sHR, sub proportional hazard ratio; NOS, not otherwise specified; cm, centimeter; mm, millimeter. [file Table_5.docx]

**Supplementary Table 5 | Multivariate compete-risk model of characteristics associated with cancer-specific survival for patients treated with SLNB and NA**

| **Characteristics** | **Origin cohort** | | **IPW cohort** | |
| --- | --- | --- | --- | --- |
|  | Unadjusted  sHR(95%CI) | *P* | Adjusted  sHR(95%CI) | *P* |
| **Region** |  |  |  |  |
| East | Reference |  | Reference |  |
| Northern Plains | 0.51 (0.19-1.40) | 0.190 | 0.41 (0.14-1.20) | 0.105 |
| Pacific Coast | 0.99 (0.61-1.62) | 0.973 | 1.06 (0.61-1.86) | 0.839 |
| Southwest | 0.23 (0.04-1.40) | 0.111 | 0.22 (0.03-1.81) | 0.159 |
| **Insurance** |  |  |  |  |
| Insured | Reference |  | Reference |  |
| Medicaid | 0.85 (0.38-1.91) | 0.689 | 0.70 (0.26-1.87) | 0.477 |
| Uninsured | 114 (0.23-5.73) | 0.878 | 1.19 (0.23-6.33) | 0.835 |
| Unknown | 1.73 (0.97-3.11) | 0.066 | 1.96 (0.99-3.87) | 0.054 |
| **Year of diagnosis** |  |  |  |  |
| 2004-2009 | Reference |  | Reference |  |
| 2010-2016 | 0.90 (0.49-1.65) | 0.731 | 0.89 (0.43-1.84) | 0.756 |
| **Age, year** |  |  |  |  |
| 18-49 | Reference |  | Reference |  |
| 50-59 | 1.44 (0.64-3.22) | 0.380 | 1.26 (0.51-3.14) | 0.620 |
| 60-69 | 1.87 (0.82-4.22) | 0.134 | 1.95 (0.78-4.87) | 0.155 |
| 70-80 | 5.48 (2.63-11.45) | **<0.001** | 7.34 (3.37-15.99) | **<0.001** |
| **Race** |  |  |  |  |
| White | Reference |  | Reference |  |
| Black | 1.22 (0.57-2.63) | 0.602 | 0.86 (0.36-2.08) | 0.740 |
| Other | 0.54 (0.16-1.85) | 0.327 | 0.85 (0.19-3.81) | 0.527 |
| **Marital status** |  |  |  |  |
| Married | Reference |  | Reference |  |
| Single | 0.88 (0.46-1.71) | 0.716 | 0.67 (0.33-1.36) | 0.270 |
| Divorced/separated/widowed | 0.84 (0.48-1.44) | 0.521 | 0.55 (0.30-1.00) | 0.051 |
| Unknown | 1.25 (0.52-2.99) | 0.614 | 0.47 (0.16-1.41) | 0.179 |
| **Primary site** |  |  |  |  |
| Labium majus | Reference |  | Reference |  |
| Labium minus | 0.32 (0.06-1.68) | 0.176 | 0.16 (0.03-0.85) | **0.032** |
| Clitoris | 1.27 (0.14-11.16) | 0.831 | 0.94 (0.10-8.40) | 0.953 |
| Overlapping lesion | 0.72 (0.24-2.17) | 0.562 | 0.26 (0.07-0.98) | **0.047** |
| Vulva, NOS | 0.64 (0.30-1.37) | 0.254 | 0.47 (0.21-1.05) | 0.066 |
| **Pathology grade** |  |  |  |  |
| Grade I | Reference |  | Reference |  |
| Grade II | 1.50 (0.87-2.61) | 0.146 | 1.94 (1.04-3.60) | **0.036** |
| Grade III/IV | 1.86 (0.93-3.71) | **0.078** | 2.33 (1.08-5.02) | **0.031** |
| Unknown | 0.58 (0.27-1.25) | 0.164 | 0.45 (0.18-1.08) | 0.074 |
| **Tumor size, cm** |  |  |  |  |
| <2 | Reference |  | Reference |  |
| 2-4 | 2.75 (1.53-4.92) | **0.001** | 4.45 (1.51-7.80) | **<0.001** |
| ≥4 | 3.23 (1.74-6.01) | **<0.001** | 3.91 (1.71-8.91) | **0.001** |
| Unknown | 1.40 (0.68-2.91) | 0.471 | 1.78 (0.68-4.63) | 0.239 |
| **Invasion depth, mm** |  |  |  |  |
| ≤1 | Reference |  | Reference |  |
| ＞1 | 3.08 (1.45-6.57) | **0.004** | 1.97 (0.98-3.97) | **0.058** |
| Unknown | 2.00 (0.93-4.29) | 0.076 | 1.53 (0.70-3.35) | 0.287 |
| **Surgery** |  |  |  |  |
| LTE | Reference |  | Reference |  |
| SV | 1.23 (0.67-2.23) | 0.502 | 1.78 (0.94-3.36) | 0.078 |
| TV | 2.69 (1.42-5.12) | **0.002** | 3.41 (1.64-7.12) | **0.001** |
| RV | 1.85 (0.92-3.72) | 0.082 | 2.98 (1.32-6.32) | **0.008** |
| **Radiotherapy** |  |  |  |  |
| No | Reference |  | Reference |  |
| Yes | 2.08 (1.20-3.57) | **0.008** | 2.13 (1.20-3.77) | **0.009** |
| **Treatment** |  |  |  |  |
| NA | Reference |  | Reference |  |
| SLNB | 0.56 (0.31-1.00) | **0.049** | 0.40 (0.23-0.70) | **0.001** |

*Abbreviations: LN-, negative regional lymph node findings; SLNB, sentinel lymph node biopsy; NA, no regional lymph node removed; IPW, inverse probability weighting; sHR, sub proportional hazard ratio; NOS, not otherwise specified; cm, centimeter; mm, millimeter*
