# Supplementary material for: Safety and Benefit Of Sentinel Lymph Nodes Biopsy Compared to Regional Lymph Node Dissection in Primary Vulvar Cancer Patients Without Distant Metastasis and Adjacent Organ Invasion: A Retrospective Population Study
Source: Front Oncol. 2021 Jul 26;11:676038. doi: 10.3389/fonc.2021.676038 (PMC8350928; doi:10.3389/fonc.2021.676038)
Supplement: Supplementary Table 6 — Multivariate accelerate failure time analysis of characteristics associated with overall survival for patients treated with SLNB and NA. LN−, negative regional lymph node findings; SLNB, sentinel lymph node biopsy; NA, no regional lymph node removed; IPW, inverse probability weighting; TR, time ratio; NOS, not otherwise specified; cm, centimeter; mm, millimeter. [file Table_6.docx]

**Supplementary Table 6 | Multivariate accelerate failure time model of characteristics associated with overall survival in the LN- cohort for patients treated with SLNB and NA**

| **Characteristics** | **ORIGIN cohort** | | **IPTW cohort** | |
| --- | --- | --- | --- | --- |
|  | Unadjusted  TR(95%CI) | *P* | Adjusted  TR(95%CI) | *P* |
| **Region** |  |  |  |  |
| East | Reference |  | Reference |  |
| Northern Plains | 1.55 (0.91-2.64) | 0.107 | 0.75 (0.36-1.56) | 0.449 |
| Pacific Coast | 1.42 (1.04-1.93) | 0.031 | 1.13 (0.81-1.58) | 0.482 |
| Southwest | 1.43 (0.72-2.83) | 0.310 | 1.68 (0.72-3.94) | 0.230 |
| **Insurance** |  |  |  |  |
| Insured | Reference |  | Reference |  |
| Medicaid | 0.83 (0.52-1.32) | 0.426 | 1.09 (0.67-1.78) | 0.720 |
| Uninsured | 1.39 (0.58-3.33) | 0.460 | 1.71 (0.70-4.16) | 0.240 |
| Unknown | 0.75 (0.52-1.07) | 0.109 | 1.06 (0.69-1.63) | 0.790 |
| **Year of diagnosis** |  |  |  |  |
| 2004-2009 | Reference |  | Reference |  |
| 2010-2016 | 1.27 (0.90-1.80) | 0.171 | 1.69 (1.07-2.67) | **0.025** |
| **Age, year** |  |  |  |  |
| 18-49 | Reference |  | Reference |  |
| 50-59 | 0.73 (0.47-1.14) | 0.166 | 0.53 (0.32-0.88) | **0.014** |
| 60-69 | 0.54 (0.35-0.83) | **0.005** | 0.54 (0.35-0.82) | **0.004** |
| 70-80 | 0.17 (0.11-0.26) | **<0.001** | 0.17 (0.10-0.31) | **<0.001** |
| **Race** |  |  |  |  |
| White | Reference |  | Reference |  |
| Black | 0.65 (0.42-1.01) | **0.054** | 0.29 (0.14-0.60) | **0.001** |
| Other | 1.45 (0.63-3.37) | 0.383 | 1.03 (0.43-2.48) | 0.941 |
| **Marital status** |  |  |  |  |
| Married | Reference |  | Reference |  |
| Single | 0.60 (0.40-0.89) | **0.012** | 1.05 (0.65-1.70) | 0.836 |
| Divorced/separated/widowed | 0.65 (0.47-0.91) | **0.012** | 0.76 (0.52-1.11) | 0.158 |
| Unknown | 0.50 (0.28-0.90) | **0.020** | 1.00 (0.47-2.13) | 0.988 |
| **Primary site** |  |  |  |  |
| Labium majus | Reference |  | Reference |  |
| Labium minus | 1.97 (0.79-4.90) | 0.146 | 2.60 (1.21-5.60) | **0.014** |
| Clitoris | 0.70 (0.21-2.36) | 0.638 | 0.68 (0.22-2.11) | 0.501 |
| Overlapping lesion | 1.20 (0.56-2.60) | 0.638 | 0.82 (0.42-1.59) | 0.552 |
| Vulva, NOS | 1.24 (0.71-2.18) | 0.450 | 1.19 (0.71-2.00) | 0.506 |
| **Pathology grade** |  |  |  |  |
| Grade I | Reference |  | Reference |  |
| Grade II | 0.62 (0.42-0.91) | **0.014** | 0.79 (0.54-1.14) | 0.206 |
| Grade III/IV | 0.64 (0.38-1.08) | **0.096** | 0.86 (0.52-1.44) | 0.568 |
| Unknown | 1.04 (0.71-1.51) | 0.858 | 1.53 (0.82-2.87) | 0.180 |
| **Tumor size, cm** |  |  |  |  |
| <2 | Reference |  | Reference |  |
| 2-4 | 0.44 (0.31-0.64) | **0.001** | 0.46 (0.30-0.71) | **0.001** |
| ≥4 | 0.34 (0.23-0.52) | **<0.001** | 0.50 (0.30-0.84) | **0.009** |
| Unknown | 0.82 (0.55-1.21) | 0.319 | 1.19 (0.74-1.90) | 0.475 |
| **Invasion depth, mm** |  |  |  |  |
| ≤1 | Reference |  | Reference |  |
| ＞1 | 0.72 (0.49-1.06) | **0.093** | 1.38 (0.95-2.02) | 0.093 |
| Unknown | 1.04 (0.73-1.50) | 0.818 | 1.70 (1.02-2.83) | **0.043** |
| **Surgery** |  |  |  |  |
| LTE | Reference |  | Reference |  |
| SV | 1.16 (0.82-1.65) | 0.394 | 0.95 (0.63-1.44) | 0.810 |
| TV | 1.00 (0.62-1.63) | 0.989 | 0.99 (0.59-1.65) | 0.966 |
| RV | 0.79 (0.50-1.25) | 0.314 | 0.66 (0.39-1.11) | 0.117 |
| **Radiotherapy** |  |  |  |  |
| No | Reference |  | Reference |  |
| Yes | 0.60 (0.39-0.91) | **0.015** | 0.79 (0.50-1.23) | 0.291 |
| **Treatment** |  |  |  |  |
| NA | Reference |  | Reference |  |
| SLNB | 1.44 (0.94-2.21) | **0.091** | 1.15 (0.76-1.73) | 0.279 |

*Abbreviations: LN-, negative regional lymph node findings; SLNB, sentinel lymph node biopsy; NA, no regional lymph node removed; IPW, inverse probability weighting; TR, time ratio; NOS, not otherwise specified; cm, centimeter; mm, millimeter*
