# Supplementary material for: Safety and Benefit Of Sentinel Lymph Nodes Biopsy Compared to Regional Lymph Node Dissection in Primary Vulvar Cancer Patients Without Distant Metastasis and Adjacent Organ Invasion: A Retrospective Population Study
Source: Front Oncol. 2021 Jul 26;11:676038. doi: 10.3389/fonc.2021.676038 (PMC8350928; doi:10.3389/fonc.2021.676038)
Supplement: Supplementary Table 7 — Effect of SLNB versus RLND from IPW adjusted multivariate AFT and CR models for sensitivity analysis about missing lymph node size. SLNB, sentinel lymph node biopsy; RLND, regional lymph node removed; IPW, inverse probability weighting; AFT, accelerate failure time; CR, compete-risk; TR, time ratio; sHR, sub proportional hazard ratio; LN+, positive regional lymph node findings; LN−, negative regional lymph node findings. [file Table_7.docx]

**Supplementary Table 7 | Effect of SLNB versus RLND from IPW adjusted multivariate AFT and CR models for sensitivity analysis about missing lymph node size**

| **Situations** | **Overall survival**  **Adjusted TR of SLNB**  **(95% CI)** | | **Cancer-specific survival**  **Adjusted sHR of SLNB (95% CI)** | |
| --- | --- | --- | --- | --- |
| **LN+ cohort** |  |  |  |  |
| Missing size as <5 mm | 2.69 (1.72-4.20) | <0.001 | 0.29 (0.16-0.54) | <0.001 |
| Missing size as ≥5 mm | 2.60 (1.68-4.00) | <0.001 | 0.31 (0.17-0.55) | <0.001 |
| **LN+ plus LN- cohort** |  |  |  |  |
| Missing size as <5 mm | 1.52 (1.03-2.24) | 0.034 | 0.43 (0.26-0.73) | 0.002 |
| Missing size as ≥5 mm | 1.58 (1.07-2.34) | 0.022 | 0.41 (0.24-0.69) | 0.001 |

*Abbreviations: SLNB, sentinel lymph node biopsy; RLND, regional lymph node removed; IPW, inverse probability weighting; AFT, accelerate failure time; CR, compete-risk; TR, time ratio; sHR, sub proportional hazard ratio; LN*+*, positive regional lymph node findings; LN-, negative regional lymph node findings*
